# Tailored phage cocktail with resistance management for controlling *Dickeya solani* in potatoes

**DOI:** 10.3389/fmicb.2026.1748314

**Published:** 2026-01-27

**Authors:** Miloud Sabri, Khaoula Mektoubi, Khaoula Habbadi, Orges Cara, Kaoutar El Handi, Angelo De Stradis, Toufic Elbeaino

**Affiliations:** 1 International Centre for Advanced Mediterranean Agronomic Studies (CIHEAM of Bari), Bari, Italy; 2 Plant Protection Research Unit, National Institute of Agronomic Research, Regional Center of Agronomic Research of Meknes INRA-CRRA, Meknes, Morocco; 3 Department of Soil, Plant and Food Science, University of Bari, Bari, Italy; 4 National Research Council of Italy (CNR), Institute for Sustainable Plant Protection (IPSP), Bari, Italy; 5 National Research Council of Italy (CNR), Institute for Sustainable Plant Protection (IPSP), Naples, Italy

**Keywords:** biocontrol, myovirus, phage resistance, potato soft rot, sequential isolation strategy

## Abstract

*Dickeya solani*, the causal agent of soft rot and blackleg diseases in potatoes, is responsible for substantial economic losses worldwide, with no effective environmentally acceptable chemical control measures available. Bacteriophage-based biocontrol represents an environmentally sustainable alternative to chemical treatments; however, the rapid emergence of resistant bacterial strains remains a major limitation to their efficacy. In this study, we developed a phage cocktail consisting of three bacteriophages (LMST, PDS1, and PDS2) through a rational, sequential isolation strategy to effectively target *D. solani* and its emerging phage-resistant strains. High-throughput sequencing and genomic analyses revealed that LMST, PDS1, and PDS2 possess double-stranded DNA genomes of 153,878, 254,449, and 133,665 bp, respectively, and lack genes associated with lysogeny, virulence, or antibiotic resistance. Transmission electron microscopy (TEM) analyses revealed a myovirus-like morphology and confirmed efficient replication of the phages on *D. solani* cells. Host range assays demonstrated that the phages were specific to *D. solani* strains, with LMST additionally infecting *D. fangzhongdai*, while thermal stability tests indicated that all three phages maintained infectivity across a broad temperature range (−20 °C to 60 °C), collectively supporting their potential as suitable biocontrol agents. Phylogenetic analyses classified LMST and PDS1 within the genera *Limestonevirus* and *Salmondvirus*, respectively, whereas PDS2 represented a highly divergent lineage, expanding the anti-*Dickeya* phage repertoire. Turbidity assays revealed that the phage cocktail more effectively suppresses *D. solani* growth than individual phages and substantially reduces the development of phage resistance. Furthermore, preventive and curative applications of the phage cocktail resulted in 68.75 and 59% reductions in *D. solani*-induced symptom severity in potato tubers, respectively. This study demonstrates that a rationally designed phage cocktail can significantly control *D. solani* infections while mitigating the emergence of phage-resistant strains.

## 1 Introduction

The potato (*Solanum tuberosum*), which originated in the Andean region of South America, is a globally significant staple food crop due to its high yield and nutritional value (Chandrasekara and Josheph Kumar, 2016; Hill et al., 2021). It serves as a low-cost source of dietary energy and contributes significantly to food security worldwide (Hill et al., 2021). According to the latest FAOSTAT data, world potato production reached approximately 383 million metric tons in 2023, with major contributions from China and India as the largest producers worldwide (Hu et al., 2025). However, potato production faces significant challenges, notably bacterial soft rot caused by pathogens of the Soft Rot Pectobacteriaceae (SRP) family (Ma et al., 2024). SRP pathogens are characterized by their secretion of an array of plant cell wall-degrading enzymes (PCWDEs) that macerate host tissue. This enzymatic degradation causes cell lysis, releasing nutrients that support bacterial propagation and population growth (Zhou et al., 2024; Babińska-Wensierska et al., 2025). Among members of SRP, *Dickeya solani* (*D. solani*) is considered the most aggressive species and is ranked among the top ten most damaging plant pathogenic bacteria globally (Dobhal et al., 2024; Grupa-Urbańska et al., 2025). The emergence of *D. solani* in Europe has resulted in substantial economic losses in potato production and is a major cause of seed lot rejection during certification processes (Toth et al., 2011; Osdaghi, 2022). Potato is the main host of *D. solani*; however, it also infects ornamental plants, such as hyacinth (*Hyacinthus orientalis*), and colonizes various common weeds, including *Cyperus rotundus*, *Urtica urens*, and *Viola arvensis* (Matilla et al., 2023). Currently, there are no commercially available potato cultivars resistant to *D. solani*, and management relies primarily on the use of certified *D. solani*-free seeds and prophylactic measures (Lebecka et al., 2021; Matilla et al., 2023). This limitation underscores the serious need for innovative management strategies. Among these, virulent (lytic) bacteriophages have demonstrated significant potential for the control of soft rot disease (Adriaenssens et al., 2012; Miroshnikov et al., 2021; Elhalag et al., 2024; Kmoch et al., 2024). Lytic phages, which are viruses that specifically infect and lyse bacteria, have many advantageous characteristics, including high host-bacteria specificity, ease of discovery, self-replicating nature, low environmental impact, cost-effectiveness, and the ability to multiply after application by targeting and eliminating bacterial hosts, unlike conventional antimicrobial compounds that tend to degrade over time (Sabri et al., 2024). However, bacteria can quickly evolve resistance to bacteriophages, limiting their effectiveness and preventing the complete eradication of a susceptible bacterial population (Oromí-Bosch et al., 2023). Phage resistance is a major obstacle to effective phage therapy, even though phage-resistant bacteria often pay fitness costs, such as reduced virulence, impaired colonization, or even renewed sensitivity to antibiotics (Mangalea and Duerkop, 2020). Several strategies are employed to combat bacterial resistance to phages, such as the use of tailored phage cocktails, which reduce the potential for cross-resistance development (Costa et al., 2024). However, most existing approaches either focus on single-step phage selection or do not systematically address the emergence of resistance during the cocktail design process. In contrast, this study presents a stepwise, resistance-informed phage cocktail strategy for controlling *D. solani*, combining sequential phage isolation, characterization, and cocktail assembly to anticipate and limit bacterial resistance. This approach offers a practical framework for potato phage therapy while providing novel insights into resistance management.

## 2 Materials and methods

### 2.1 Bacterial strains and culture conditions

*Dickeya solani* strain CFBP 7373, obtained from the “French Collection of Phytopathogenic Bacteria” (Angers, France), was used throughout this study. The strains listed in Table 1 were grown at 28 °C either in liquid yeast extract peptone glucose broth (YPG) (5.0 g/L yeast extract, 5.0 g/L peptone and 10.0 g/L glucose) or on yeast extract peptone glucose agar (YPGA, i.e., YPG supplemented with 1.5% agar). For long-term storage, the strains were stored at −80 °C in 25% glycerol prepared in YPG broth.

**Table 1:** Bacterial strains used for determining the host range of isolated phages.

Species	Strain ID	Isolation source	Geographic origin
*Dickeya solani*	CFBP 7373	*Solanum tuberosum*	Syria
*Dickeya solani*	CFBP 7374	*Solanum tuberosum*	Syria
*Dickeya solani*	CFBP 5647	*Lycopersicon esculentum*	France
*Dickeya solani*	CFBP 7085	*Solanum tuberosum*	Spain
*Dickeya solani*	CFBP 7345	*Solanum tuberosum*	Netherlands
*Dickeya solani*	CFBP 8199	*Solanum tuberosum*	Netherlands
*Dickeya solani*	CFBP 8929	water	France
*Dickeya solani*	CFBP 8936	water	France
*Dickeya solani*	CFBP 8940	water	France
*Dickeya solani*	CFBP 8941	water	France
*Dickeya fangzhongdai*	C7**	*Brassica oleracea* var. *botrytis*	Italy
*Pseudomonas savastanoi* pv. *savastanoi*	CFBP 7006	*Olea europaea*	Syria
*Xanthomonas fragariae*	CFBP 5253	*Fragaria* sp.	France
*Xanthomonas euvesicatoria*	CFBP 6807	-	USA
*Xanthomonas vesicatoria*	CFBP 7996	*Solanum lycopersicum*	France
*Xanthomonas perforans*	CFBP 7993	*Solanum lycopersicum*	Mauritius
*Xanthomonas gardneri*	CFBP 8588	*Solanum lycopersicum*	France
*Agrobacterium tumefaciens*	BPIC 284	*Prunus dulcis*	Greece
*Pseudomonas fluorescens*	CFBP 2392	*Phaseolus vulgaris*	France
*Leuconostoc mesenteroides*	MS4	water	Morocco
*Lactococcus lactis* subsp. *lactis*	ATCC 11454	-	USA
*Pseudomonas hunanensis*	B10**	*Brassica oleracea var. italica*	Italy
*Pseudomonas putida*	B14**	*Brassica oleracea var. italica*	Italy
*Pseudomonas fulva*	C29**	*Brassica oleracea var. botrytis*	Italy
*Pseudomonas graminis*	C23**	*Brassica oleracea var. botrytis*	Italy
*Bacillus siamensis*	C36**	*Brassica oleracea var. botrytis*	Italy
*Bacillus subtilis*	C70**	*Brassica oleracea var. botrytis*	Italy
*Pantoea agglomerans*	C6**	*Brassica oleracea var. botrytis*	Italy

**Collection of the Laboratory of Bacteriology and Phage Biocontrol, CIHEAM-IAM, Bari, Italy.

CFBP, French Collection of Phytopathogenic Bacteria, Angers, France. BPIC, Benaki Phytopathological Institute collection.

### 2.2 Step-by-step isolation of bacteriophages targeting emerging resistant strains of *Dickeya solani*

The bacteriophages analyzed in this study were isolated from three sewage water samples collected in October 2024 and January 2025 in Souk Sabt Jahjouh, Meknes-Fès region, Morocco (33°42′52″N, 5°39′05″W). Each of the three sewage water samples was processed individually as follows: 500 mL of each sample was first filtered using Grade 1 filter paper (Whatman, 75 × 100 mm) to remove large debris. The filtrates were subsequently passed through 0.22 μm syringe filters (Acrodisc®, Merck, Rome) to eliminate bacterial cells and fine debris. For phage enrichment, 2 mL of the filtrate of the first sample was mixed with 2 mL of an overnight culture of *D. solani* CFBP 7373 (10^8^ CFU/mL). The mixture was added to 10 mL of YPG broth and incubated at 28 °C for 24 h under agitation at 120 rpm. Following incubation, the culture supernatant containing amplified phages was clarified by 0.22 μm filtration and stored at 4 °C for further analysis. A single phage, designated LMST, was isolated and purified using the double agar overlay method (Kropinski et al., 2009). To induce the emergence of phage-resistant bacterial strains, *D. solani* CFBP 7373 was co-cultivated with phage LMST in YPG broth at 28 °C for 72 h. The reappearance of bacterial growth in the presence of phage indicated the development of resistance. After 72 h, ten resistant strains were isolated and purified on YPGA plates, and their resistance was confirmed using the spot-on-lawn assay (Sabri et al., 2025). These phage-resistant strains were subsequently employed as hosts for the isolation of a second phage, designated PDS1, through a repeated enrichment procedure using the second wastewater filtrate. The process was repeated once more, employing strains resistant to the second phage, to obtain a third distinct phage (hereafter referred to as PDS2).

For high-titer lysate preparation, 200 μL of each purified phage suspension was mixed with 200 μL of a *D. solani* culture (10^8^ CFU/mL) and inoculated into 2 mL of YPG broth. The cultures were incubated at 28 °C for 24 h, after which the suspensions were filtered through 0.22 μm filters. Phage titers were determined using the double agar overlay method, and equal volumes of the three amplified phage stocks (10^8^ PFU/mL) were subsequently combined to generate a phage cocktail for downstream applications.

### 2.3 Transmission electron microscopy and metadata analyses

The morphological features and lytic activity of the isolated phages (LMST, PDS1, and PDS2) against *D. solani* were investigated using transmission electron microscopy (TEM). Briefly, cultures of *D. solani* were challenged with LMST, PDS1, and PDS2 suspensions (multiplicity of infection (MOI) = 1) at 28 °C for 24 h. Phage suspensions or infected cells were adsorbed for 2 min onto carbon-coated copper/rhodium grids, rinsed with distilled water, and negatively stained with a 0.5% (w/v) aqueous solution of UA-Zero EM stain (Agar Scientific, UK). Grids were examined and representative images were taken via an FEI Morgagni 282D TEM (FEI, Hillsboro, OR, USA) at 80 kV.

Phages’ metadata, head diameter and tail length, were extracted from the TEM micrographs using Adobe Photoshop software for dimensional measurements and analyzed using standard Excel statistical formulas. Counts were determined on a sample of 50 units per population.

### 2.4 Phage DNA extraction and full genome sequencing

Genomic DNA of LMST, PDS1, and PDS2 was extracted using the cetyltrimethylammonium bromide (CTAB) protocol (Wilson, 2001). Complete genome sequencing was performed by Eurofins Genomics (Ebersberg, Germany), using Illumina HiSeq 2,500 paired-end sequencing technology, employing TruSeq-like library preparation workflows, with an average read length of 150 bp. Raw data were quality-filtered, trimmed, and *de novo* assembled using the Geneious Prime 2025.1.1 software (Biomatters, Ltd., San Diego, CA, USA). Genome annotation was carried out with Pharokka (Galaxy v1.3.2) (Bouras et al., 2023), which identifies predicted coding sequences (CDS), transfer RNAs (tRNAs), transfer-messenger RNAs (tmRNAs), and clustered regularly interspaced short palindromic repeats (CRISPRs). Functional annotation of CDS was performed using the PHROGs database, and protein-coding sequences were predicted with PHANOTATE. Antimicrobial resistance (AMR) and virulence genes were screened with AMRFinderPlus (Galaxy v3.12.8) (Feldgarden et al., 2021) and ABRicate (Galaxy v1.0.1) (Seemann, 2016). The phage lifestyles were inferred using PhaTYP program (Shang et al., 2023). Intergenomic similarities among phage genomes were calculated using VIRIDIC with default settings (Moraru et al., 2020), comparing the newly sequenced phages to publicly available reference genomes. The complete genome sequences of LMST, PDS1, and PDS2 were deposited at GenBank and circular maps of the genomes and phylogenetic trees were constructed using ViPTree (Nishimura et al., 2017).

### 2.5 Spot assay for phage lytic activity

To assess the bacteriolytic activity of phages LMST, PDS1, and PDS2 against *D. solani* and its emerging resistant strains, spot assays were conducted. Briefly, 200 μL of *D. solani* suspension (10^8^ CFU/mL) were mixed with 6 mL of YPG soft agar (i.e., YPG supplemented with 0.7% agar), poured into YPGA plates, and allowed to air-dry within the laminar flow hood. Subsequently, drops of phage suspension (10 μL) at different concentrations (10^8^, 10^7^, 10^6^, 10^5^, 10^4^, and 10^3^ PFU/mL) were spotted onto the surface of the plates. The plates were incubated at 28 °C for 24 h and the bacteriolytic activity of the phages was perceived as a clear inhibition zone of *D. solani* growth. All experiments were performed with three biological replicates per phage concentration and repeated three times independently.

### 2.6 Turbidity assay for quantification of phage-induced bacterial lysis

The lytic potential of phages LMST, PDS1, and PDS2 in inhibiting the growth of *D. solani* in liquid culture was evaluated both individually and in combination. Briefly, phage suspensions (200 μL) were mixed with *D. solani* suspensions (200 μL) at MOIs of 1, 0.1, 0.01, or 0.001. Each mixture was then inoculated into 2 mL of YPG broth in triplicate and incubated at 28 °C for 4 days. Optical density (OD₆₀₀) was measured at multiple time points (0, 2, 4, 6, 20, 24, 30, 48, 72, and 96 h) using a UV–Vis spectrophotometer to track bacterial growth dynamics.

### 2.7 Thermal stability of phages

The thermal stability of phages LMST, PDS1, and PDS2 was evaluated by incubating aliquots of each phage suspension (~10^8^ PFU/mL) at various temperatures (−20, 4, 28, 40, 50, 60, and 70 °C) for 24 h. Following the incubation period, serial dilutions were made with phage buffer (100 mM Tris–HCl, pH 7.6; 10 mM MgCl_2_; 100 mM NaCl; 10 mM MgSO_4_), and residual phage titers were determined using the double agar overlay method.

### 2.8 Host range determination

The host range of phages LMST, PDS1, and PDS2 was evaluated against a panel of phytopathogenic and beneficial bacterial strains (Table 1) using the spot assay as described above. Bacterial lawns were inoculated with 10 μL of each phage suspension (10^8^ to 10^5^ PFU/mL) and incubated at 28 °C for 24 h. Following incubation, the plates were examined for the presence of lytic zones, which were scored as clear (++), turbid (+), or absent (−). Bacterial strains exhibiting no signs of lysis were considered phage-insensitive.

### 2.9 Evaluation of the *ex vivo* efficacy of the designed phage cocktail in the control of soft rot disease

The efficacy of LMST, PDS1, and PDS2 in combination, in controlling soft rot disease was evaluated in potato tubers (*Solanum tuberosum* cv. Monalisa) according to Dees et al. (2017). Potato tubers were surface disinfected by immersion in 70% ethanol for 3 min, washed with sterile distilled water, and air-dried under a laminar flow hood. Circular wells (approximately 2 mm in diameter and 1 cm in depth) were created by punching the tuber surface with the tip of a sterile Pasteur pipette. For the preventive treatment, 100 μL of the phage cocktail suspension (10^8^ PFU/ml) was applied to each well. After 24 h, 100 μL of an overnight culture of *D. solani* (10^8^ CFU/mL) was inoculated into the same well. For the curative treatment, the wells were inoculated with 100 μL of *D. solani* (10^8^ CFU/mL), and after 24 h, 100 μL of the cocktail suspension was applied. Untreated tubers inoculated with sterile water or with *D. solani* only were included as negative and positive controls, respectively. The inoculated tubers were subsequently placed in sterile, high-humidity chambers and incubated at 28 °C for 7 days. For each treatment group, ten tubers were used, and the experiment was independently repeated twice under the same conditions. After incubation, each tuber was cut vertically through the inoculation points and soft rot symptoms were quantified. The experiment was carried out twice and results were quantified by measuring the area of macerated tissue per treatment group according to an arbitrary six-level disease scale whereby: 0: No symptoms; 1: Slight maceration (1–10% of tuber volume); 2: Moderate maceration (11–25%); 3: Severe maceration (26–50%); 4: Extensive maceration (51–75%); 5: Complete maceration (>75%). The efficacy of each treatment in controlling soft rot disease was calculated as follows:


$$\text{Efficacy}\;(\%)=100-(\frac{\sum (P\times T)}{\sum (P\times C)}\times 100)$$

Where P = severity score, T = number of treated tubers having the same score, and C = number of infected tubers (positive control) having the same score.

To quantify the *D. solani* load in treated and untreated tubers, a TaqMan quantitative PCR (qPCR) assay was conducted. Briefly, 1 g of inoculated tissue was collected from each tuber and subjected to DNA extraction using the CTAB method (Wilson, 2001). Subsequently, TaqMan-based qPCR assays were carried out in a thermocycler apparatus (Bio-Rad CFX96, BioRad, Milan, Italy), using *D. solani* specific primers ds-f: 5′-GCGAACTTCAACGGTAAA-3′ and ds-r: 5′- CAGAGCTACCAACAGAGA −3′, along with the probe ds-p: 5′-6FAM-CTCTGCTGGACGGTTC-BHQ-1-3′ as described by Vaerenbergh et al. (2012). The relative quantity of bacterial DNA in each sample was determined using the relative quantification method with an external calibrator (Livak and Schmittgen, 2001), calculated as follows:


$$\text{Relative Quantity}=2^{-\mathrm{\Delta Cq}},\mathrm{\Delta Cq}=Cq_{\text{Sample}}-Cq_{\text{Reference Standard}}$$

### 2.10 Statistical data analysis

Statistical analyses were performed using RStudio. Data normality was assessed using the Shapiro–Wilk test, which revealed a significant deviation from normality. Consequently, the nonparametric Kruskal-Wallis test was applied to compare treatments. *Post hoc* pairwise comparisons were carried out using Dunn’s test with Bonferroni correction to adjust for multiple comparisons. Statistically significant groupings were determined using the multcompLetters function. Mean values and standard errors (SE) were calculated for each treatment for graphical representation.

## 3 Results

### 3.1 Morphology and lytic potential of isolated phages

**Figure 1:**
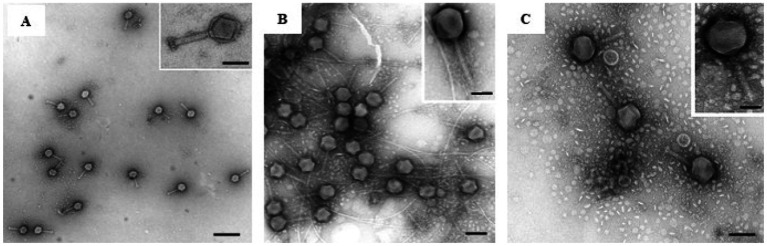
Transmission electron micrographs showing the morphology of phages: **(A)** LMST, **(B)** PDS1, and **(C)** PDS2. Scale bar = 100 nm, inset 50 nm. Bars: 100 nm **(A)**, 25 nm (inset); 80 nm **(B)**, 50 nm (inset); 100 nm **(C)**, 50 nm (inset).

Three bacteriophages capable of lysing *D. solani* were successfully isolated from sewage water samples referred to as LMST, PDS1 and PDS2. TEM analysis showed that LMST, PDS1, and PDS2 exhibited morphological characteristics typical of the myoviridae morphotype A1, featuring an isometric head, with, respectively, 85, 90,110 nm in diameter, and a contractile tail with length of 120, 130 and 140 nm (Figure 1).

**Figure 2:**
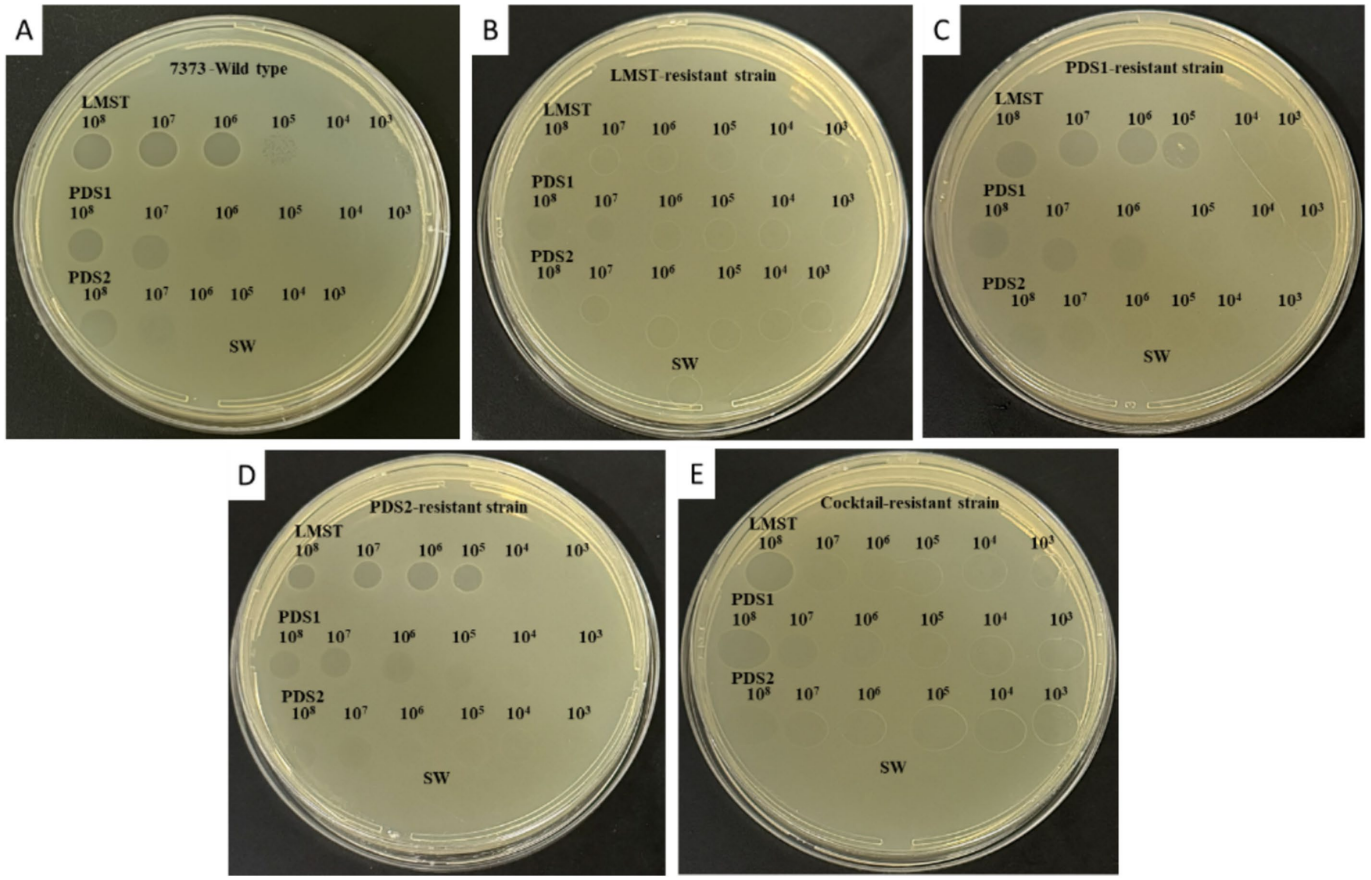
YPGA plates showing the bacteriolytic activity of phages LMST, PDS1, and PDS2 at different titers (10^8^ to 10^3^ PFU/mL) against **(A)** the wild-type *D. solani* strain CFBP 7373, **(B)** the LMST-resistant strain, **(C)** the PDS1-resistant strain, **(D)** the PDS2-resistant strain, and **(E)** the cocktail-resistant strain. Sterile water was used as a negative control.

The isolated phages demonstrated lytic activity against the wild-type *D. solani* strain CFBP 7373, producing clear plaques in spot assays (Figure 2A). LMST exhibited the highest lytic potency, effectively lysing the bacterium across a concentration range of 10^8^ to 10^5^ PFU/mL, whereas PDS1 and PDS2 lysed *D. solani* at both 10^8^ and 10^7^ PFU/mL (Figure 2A). Sequential isolation of the phages indicated that strains resistant to one phage remained susceptible to the others, suggesting a degree of complementarity among the isolated phages (Figures 2B–D). For example, strains that developed resistant to LMST were no longer lysed by this phage but retained susceptibility to PDS1 and PDS2 (Figure 2B). This pattern of specific resistance with cross-susceptibility was observed in all isolated phage-resistant strains. Interestingly, PDS1- and PDS2-derived strains remained susceptible to PDS1 and PDS2, respectively, suggesting that the resistance mechanism in these strains differs from that developed by *D. solan*i against the LMST phage (Figures 2B–D). Additionally, strains that emerged following phage cocktail treatment remained susceptible to all three phages, with reduced sensitivity noted for LMST (Figure 2E). These preliminary observations suggest that the isolated phages are distinct and exhibit complementary activity against *D. solani*, which could potentially be useful for addressing phage resistance.

### 3.2 Interaction of LMST, PDS1, and PDS2 with *Dickeya solani* cells

**Figure 3:**
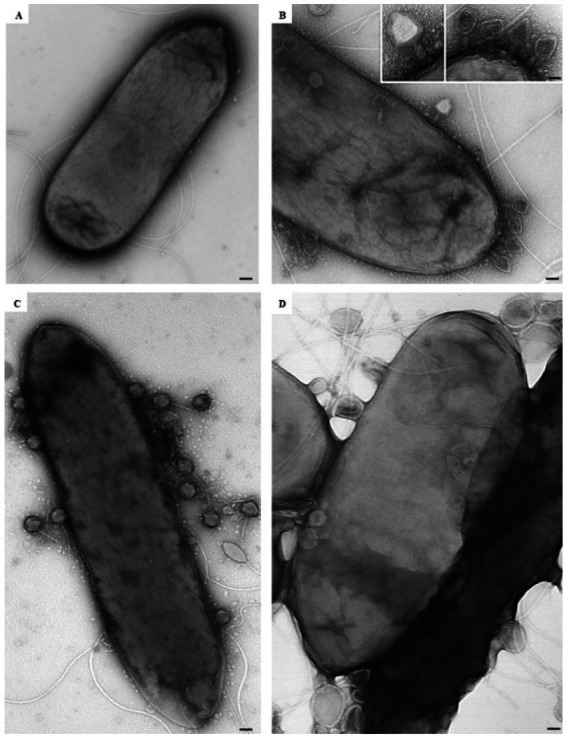
Transmission electron micrographs showing the interaction of phages LMST **(B)**, PDS1 **(C)**, and PDS2 **(D)** with *D. solani* strain CFBP 7373 **(A)**. Bars: 100 nm **(A,C,D)**; 50 nm **(B)**, 25 nm (inset).

**Figure 4:**
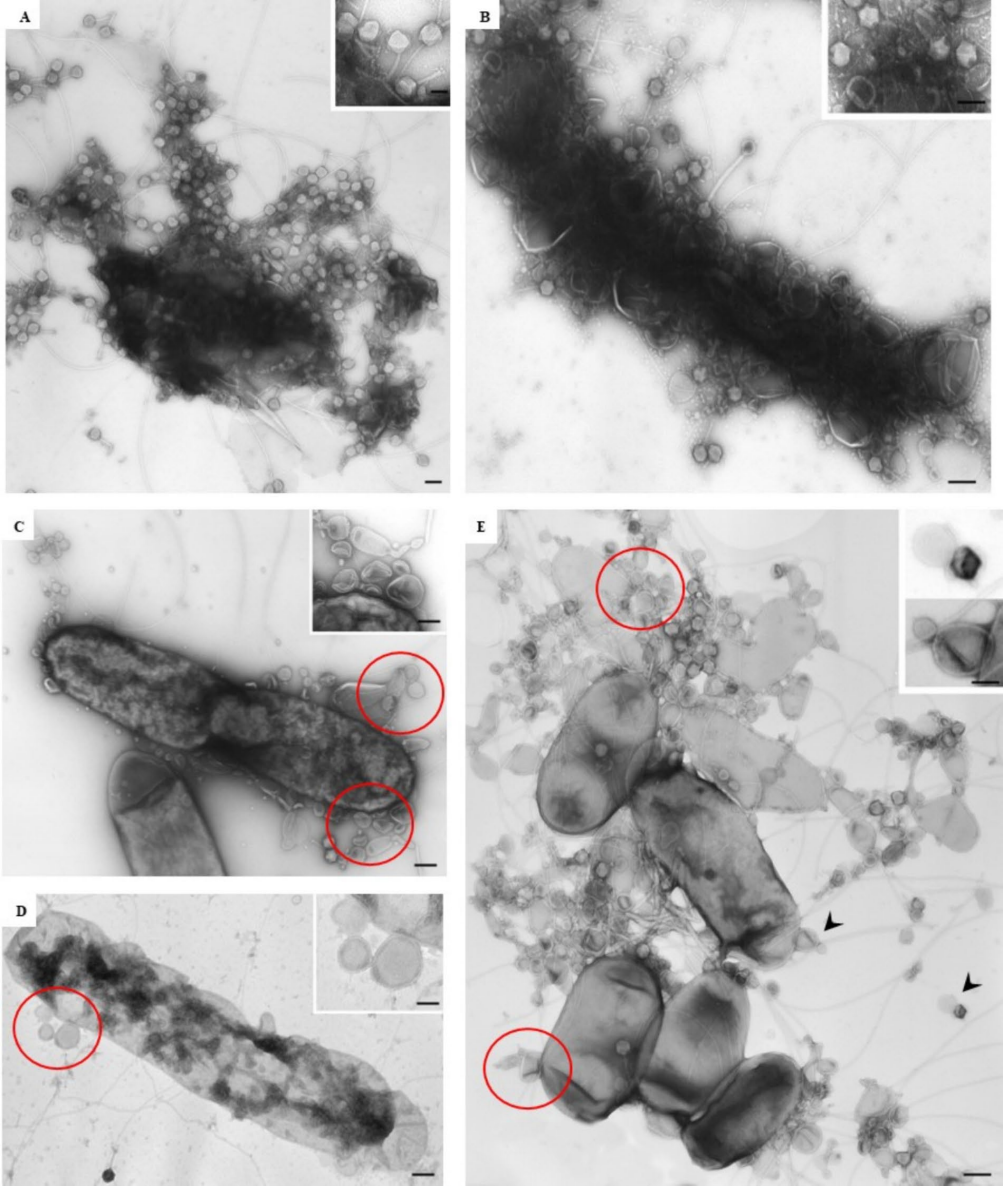
Transmission electron micrographs showing the interaction of phages LMST, PDS1, and PDS2 with *D. solani* strain CFBP 7373. **(A,B)** Bacterial lysis and phage release following infection with LMST and PDS1, respectively; **(C,D)** disruption and lysis of *D. solani* cells challenged with PDS2, accompanied by the production of outer-membrane vesicles (OMVs; red circles); **(E)** release of PDS2 phages and adsorption to OMVs (magnification of arrowhead in inset). Bars: 100 nm **(A–D)**; 250 nm **(E)**; 50 nm [inset **(A,B,D)**]; 75 nm [inset **(C)**]; 100 nm [inset **(E)**].

At the ultrastructural level, the interaction between the isolated phages and *D. solani* cells (Figure 3A) was examined to assess their lytic potential. The TEM observations revealed multiple stages of the lytic cycle of LMST, PDS1, and PDS2. The phages were observed adsorbing to the surface of *D. solani* cells (Figures 3B–D), followed by the disruption and lysis of the bacterial cells, which was accompanied by the release of progeny phages (Figures 4A,B). Interestingly, Figure 3B shows numerous LMST particles adsorbed to *D. solani* cells, in the inset (right), several particles appear darkly stained, which may correspond to empty capsids following DNA ejection, as the stain can penetrate the interior, producing a dark appearance; these empty capsids may also appear slightly collapsed or wrinkled due to loss of internal pressure. By contrast, a single LMST particle in the inset (left) appears bright, suggesting a DNA-filled capsid, where the interior excludes the stain, resulting in a clear appearance with a sharply defined capsid outline. Remarkably, challenge of *D. solani* with PDS2 resulted in the production of outer-membrane vesicles (OMVs) (Figures 4C–E red circles), a response not observed upon challenge with LMST or PDS1 (Figures 4A,B). This phenomenon represents a known bacterial defense mechanism, in which OMVs act as decoys that capture and inactivate phages (Egido et al., 2022). Correspondingly, the inset in Figure 4E shows adsorption of PDS2 phages to the OMVs produced by *D. solani*, suggesting a potential mechanism by which the bacterium prevents PDS2 particles from reaching their cellular receptors.

### 3.3 Genomic and phylogenetic analyses of phages

**Figure 5:**
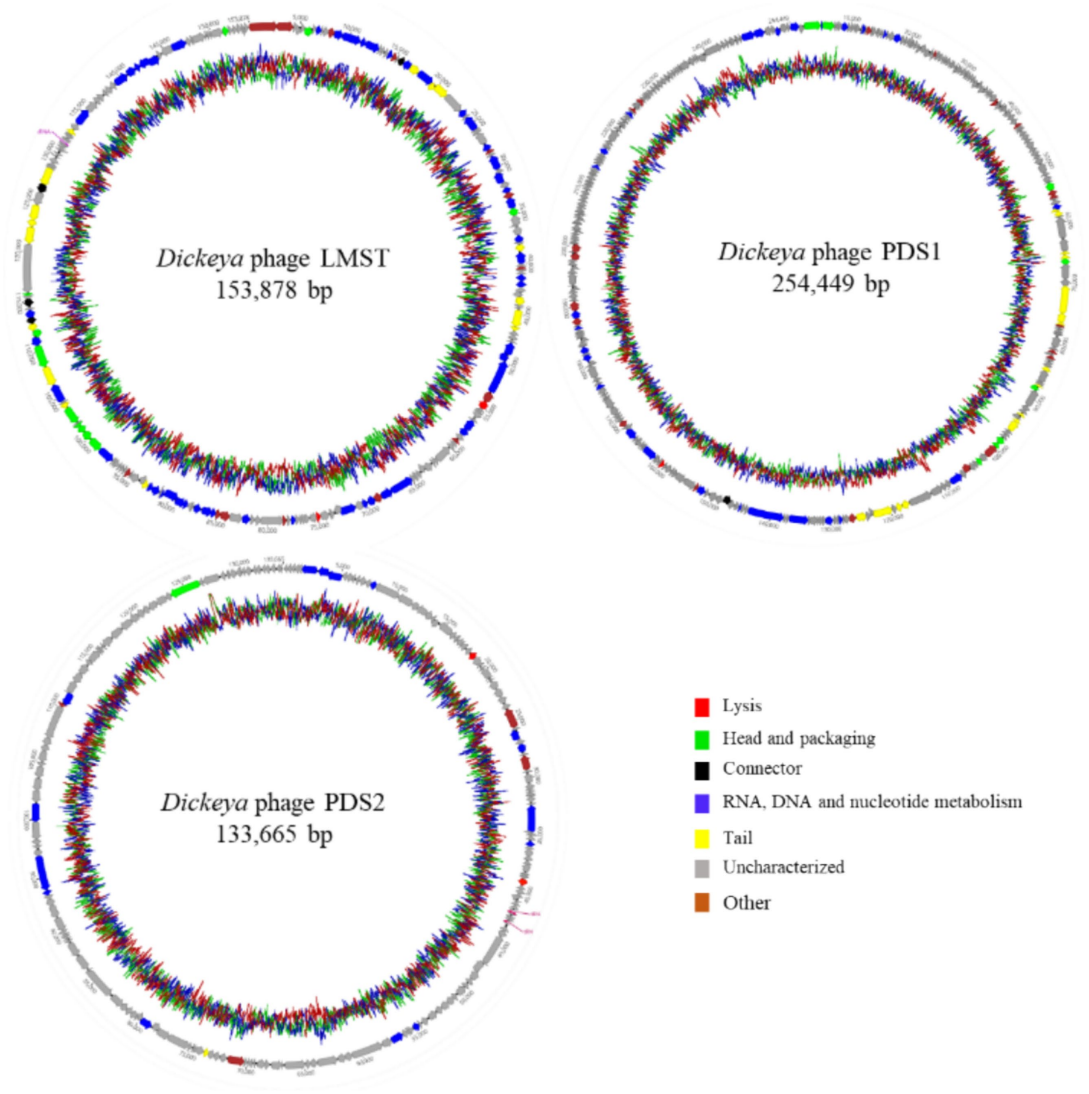
Circular genome maps of *Dickeya* phages LMST, PDS1, and PDS2 generated using Geneious Prime 2025.1.1. The inner rings illustrate the GC content percentage, while predicted coding sequences identified by Pharokka are depicted as colored arrows, with coloration reflecting their assigned functional categories.

Complete genome sequencing and *de novo* assembly of LMST, PDS1, and PDS2 revealed double-stranded DNA genomes of 153,878, 254,449, and 133,665 bp in length, respectively. The complete genome of LMST consisted of 222 coding sequences (CDS), of which the functions of 100 CDS (45.0%) were predicted; while the complete genome of PDS1 comprised 355 CDS, of which functional annotations were assigned to 75 (21.1%). The complete genome of PDS2 comprised 210 CDS, of which functional annotations could be assigned to only 24 (11.4%). Pharokka annotation of the phage genomes revealed the presence of two tRNA genes in PDS2, one tRNA gene in LMST, and no tRNA genes in PDS1. The genomic characteristics and genome maps of phages LMST, PDS1, and PDS2 are shown in Table 2 and Figure 5. PhaTYP analyses predicted that the three phages undergo a strictly lytic infection cycle, with no evidence of temperate markers, virulence factors or AMR genes. These findings were corroborated by ABRICATE and AMRFinderPlus results, which confirmed the absence of AMR and virulence genes in phage genomes. These results indicate that LMST, PDS1, and PDS2 are suitable for use as biocontrol agents.

**Table 2:** Summary of the genomic characteristics of the isolated phages.

Feature	LMST	PDS1	PDS2
Genome size (bp)	153,878	254,449	133,665
GC content (%)	49.1	44.5	46.1
Coding sequences (CDS)	222	355	210
tRNA genes	1	0	2
Lysis genes	Spanin, Endolysin	Endolysin	Endolysin 1, and 2
Lysogenic genes	0	0	0
Virulence or AMR genes	0	0	0
Hypothetical proteins	122	280	186
GenBank accession number	PX290024	PX290025	PX290026
Most similar by BLAST	Dickeya phage vB_DsoM_LIMEstone1 (NC_019925)	Dickeya phage vB_DsoM_JA11 (NC_048077)	Aeromonas phage Aes508 (NC_019543)
Identity (%)	99.38	98.52	25.01

**Figure 6:**
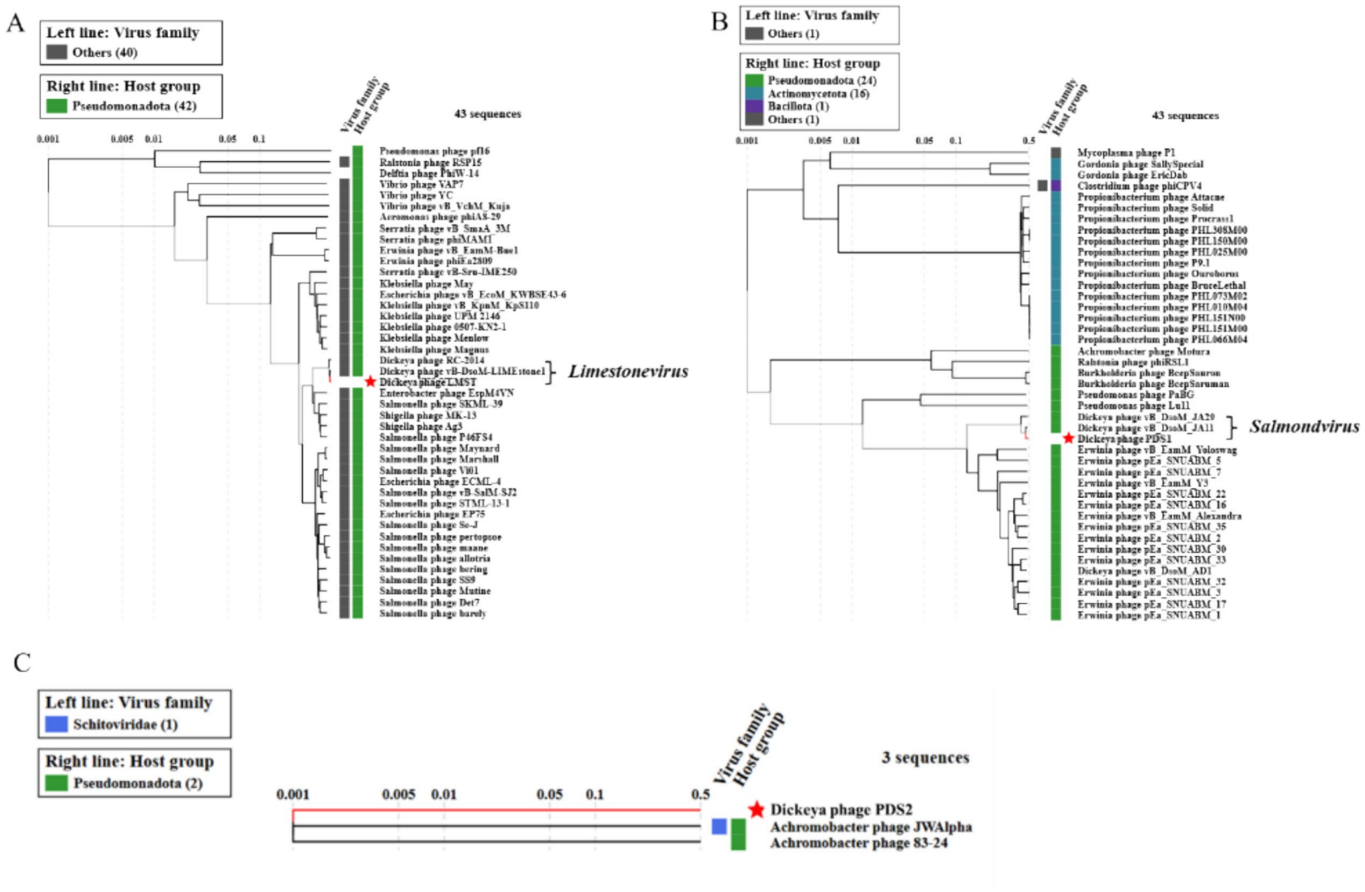
Proteomic trees of LMST **(A)**, PDS1 **(B)**, and PDS2 **(C)** generated by ViPTree using genome-wide tBLASTx similarities. LMST and PDS1 cluster within the genera *Limestonevirus* and *Salmondvirus*, respectively, while PDS2 forms a distinct lineage.

Comparative genomic analysis using BLASTn against the NCBI nucleotide database revealed that LMST is closely related to phages in the *Limestonevirus* genus (*Ackermannviridae* family), while PDS1 showed similarity to phages belonging to *Salmondvirus* genus. Both genera, *Limestonevirus* and *Salmondvirus*, comprise numerous phages known to infect *D. solani*. LMST shares the highest sequence similarity (identity: 99.38%, coverage: 98%) with *Dickeya* phage vB_DsoM_LIMEstone1 (GenBank: NC_019925), while PDS1 showed the closest homology to *Dickeya* phage vB_DsoM_JA11 (GenBank: NC_048077), with 98.52% identity and 98% coverage. Additionally, intergenomic similarity analyses using VIRIDIC revealed a value of 97.90% between phage LMST and *Dickeya* phage vB_DsoM_LIMEstone1, indicating that LMST belongs to the same species, as this value exceeds the 95% species demarcation threshold established by the “*International Committee on Taxonomy of Viruses*” (ICTV). Similarly, phage PDS1 exhibited an intergenomic similarity of 95.92% relative to *Dickeya* phage vB_DsoM_JA11, suggesting that PDS1 also likely represents the same species. In contrast, the closest hit identified for PDS2 was *Aeromonas* phage Aes508 (GenBank: NC_019543), with an intergenomic similarity of 0.12%. No other significant sequence matches were detected. These results suggest that PDS2 likely represents a novel genus or even a distinct family, highlighting its uniqueness and potential for expanding the current understanding of phage diversity. Furthermore, phylogenetic analysis based on whole-proteome comparisons, generated using ViPTree, corroborated the genomic analyses and positioned phages LMST and PDS1 alongside other *Dickeya* phages within the genera *Limestonevirus* and *Salmondvirus*, respectively (Figures 6A,B). Additionally, the ViPTree analysis placed PDS2 on a distinct branch, with only two phages showing minimal relatedness (0.001), indicating that PDS2 is highly divergent from all currently characterized phages (Figure 6C). The complete genome sequences of LMST, PDS1, and PDS2 were deposited in GenBank under the accession numbers PX290024, PX290025, and PX290026, respectively.

### 3.4 Individual and combined antibacterial effects of LMST, PDS1, and PDS2 on *Dickeya solani* growth

**Figure 7:**
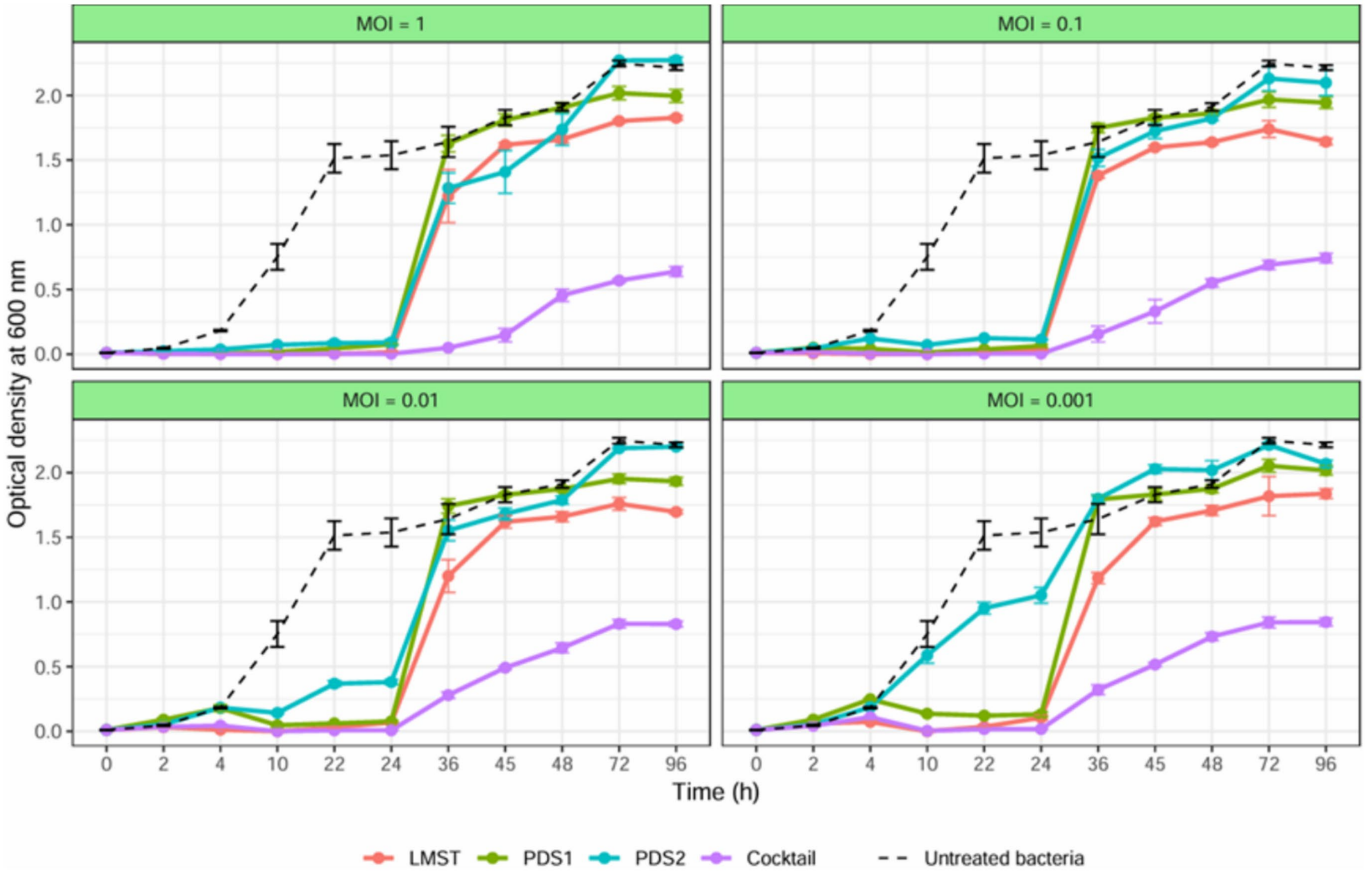
Growth curves for *Dickeya solani* strain CFBP 7373 treated with LMST, PDS1 and PDS2, individually and in combination, at different MOIs. The optical density of treated and untreated bacterial cultures for 96 h is compared. Data represents the mean ± standard deviation of three biological replicates (*n* = 3).

The antibacterial activity of bacteriophages LMST, PDS1, and PDS2 against *D. solani* in YPG broth was evaluated both individually and in combination. In untreated controls, *D. solani* exhibited a growth curve, reaching an OD₆₀₀ value greater than 1.5 after 24 h (Figure 7). In contrast, all phage treatments significantly inhibited bacterial growth during the initial 24 h, with the phage cocktail exerting the strongest inhibitory effect across all MOIs and maintaining bacterial density at minimal levels (Figure 7). Following 24 h of incubation, all phage-treated cultures exhibited a gradual increase in turbidity; however, the rate of increase was substantially lower in cultures treated with the phage cocktail. This increase continued over 96 h, with cultures challenged with individual phages reached OD₆₀₀ values exceeding 1.6, whereas those treated with the phage cocktail maintained OD₆₀₀ below 0.8, corresponding to inhibition rates exceeding 64%. Regrowth in the phage-treated cultures was attributed to the emergence of phage-resistant strains, as confirmed by spot assays. Taken together, these findings demonstrate that the phage cocktail achieved superior control of *D. solani* growth relative to single-phage treatments and effectively mitigated the development of phage resistance. The enhanced antibacterial performance of the cocktail supports the existence of synergistic interactions among the component phages and underscores their potential application as a promising biocontrol strategy for *D. solani* management.

### 3.5 Thermal stability of phages

Thermal stability assays revealed that all three phages retained infectivity across a wide temperature range (−20 °C to 60 °C), exhibiting maximal stability at 4 °C (Supplementary Figure 1). However, a complete loss of infectivity was observed for LMST at 70 °C. Phages PDS1 and PDS2 exhibited higher thermal stability, retaining activity at 70 °C, with PDS2 showing no statistically significant difference in titer between 50 °C and 70 °C. PDS1 demonstrated the greatest thermal stability, with no significant decline in titer from 25 °C to 60 °C (Supplementary Figure 1). These findings underscore the potential of these phages for application across a range of biotechnological and agricultural contexts.

### 3.6 Host range determination

Host range analysis showed that LMST, PDS1, and PDS2 were able to lyse all 10 tested *D. solani* strains (Table 3), which were obtained from the CFBP collection and originate from diverse sources across four countries (Table 1). Among the tested strains, LMST, PDS1, and PDS2 showed the strongest lytic activity against CFBP 7373 and CFBP 7374, which may be regarded as their preferred hosts under the conditions tested. Notably, LMST displayed the broadest spectrum of activity and was the only phage capable of lysing *D. fangzhongdai*, indicating a wider host range within *Dickeya* species. No lytic activity was observed against other tested plant pathogenic or beneficial bacteria, suggesting that all three phages exhibit high specificity for *Dickeya* strains and highlighting their potential as targeted biocontrol agents.

**Table 3:** Host range analysis shows the bacteriolytic effects of bacteriophages LMST, PDS1, and PDS2 at different titers (10^8^–10^5^ PFU/mL) against *Dickeya* strains.

Strain/Phage	10^8^ PFU/mL			10^7^ PFU/mL			10^6^ PFU/mL			10^5^ PFU/mL		
	LMST	PDS1	PDS2	LMST	PDS1	PDS2	LMST	PDS1	PDS2	LMST	PDS1	PDS2
*D*. *solani* CFBP 7373	++	++	++	++	++	++	++	+	+	++	+	+
*D*. *solani* CFBP 7374	++	++	++	++	++	++	++	+	+	++	+	+
*D*. *solani* CFBP 8199	++	++	+	++	+	+	+	+	+	+	+	+
*D*. *solani* CFBP 7345	++	++	++	++	+	+	++	−	−	++	−	−
*D*. *solani* CFBP 7085	++	+	+	++	+	+	++	+	+	++	+	+
*D*. *solani* 8,936	++	++	++	++	+	+	+	+	+	−	−	−
*D*. *solani* CFBP 8929	++	++	++	++	+	+	+	−	−	−	−	−
*D*. *solani* CFBP 8940	++	+	++	++	+	+	+	+	+	−	−	−
*D*. *solani* CFBP 8941	++	++	++	++	+	+	+	+	−	−	−	−
*D*. *solani* CFBP 5647	++	++	++	++	+	+	+	−	−	−	−	−
*D*. *fangzhongdai* C7	++	−	−	++	−	−	+	−	−	−	−	−

Results are scored as clear lysis (++), turbid lysis (+), or no lysis (−).

### 3.7 *Ex vivo* efficacy of phage cocktail in the control of soft rot disease

**Figure 8:**
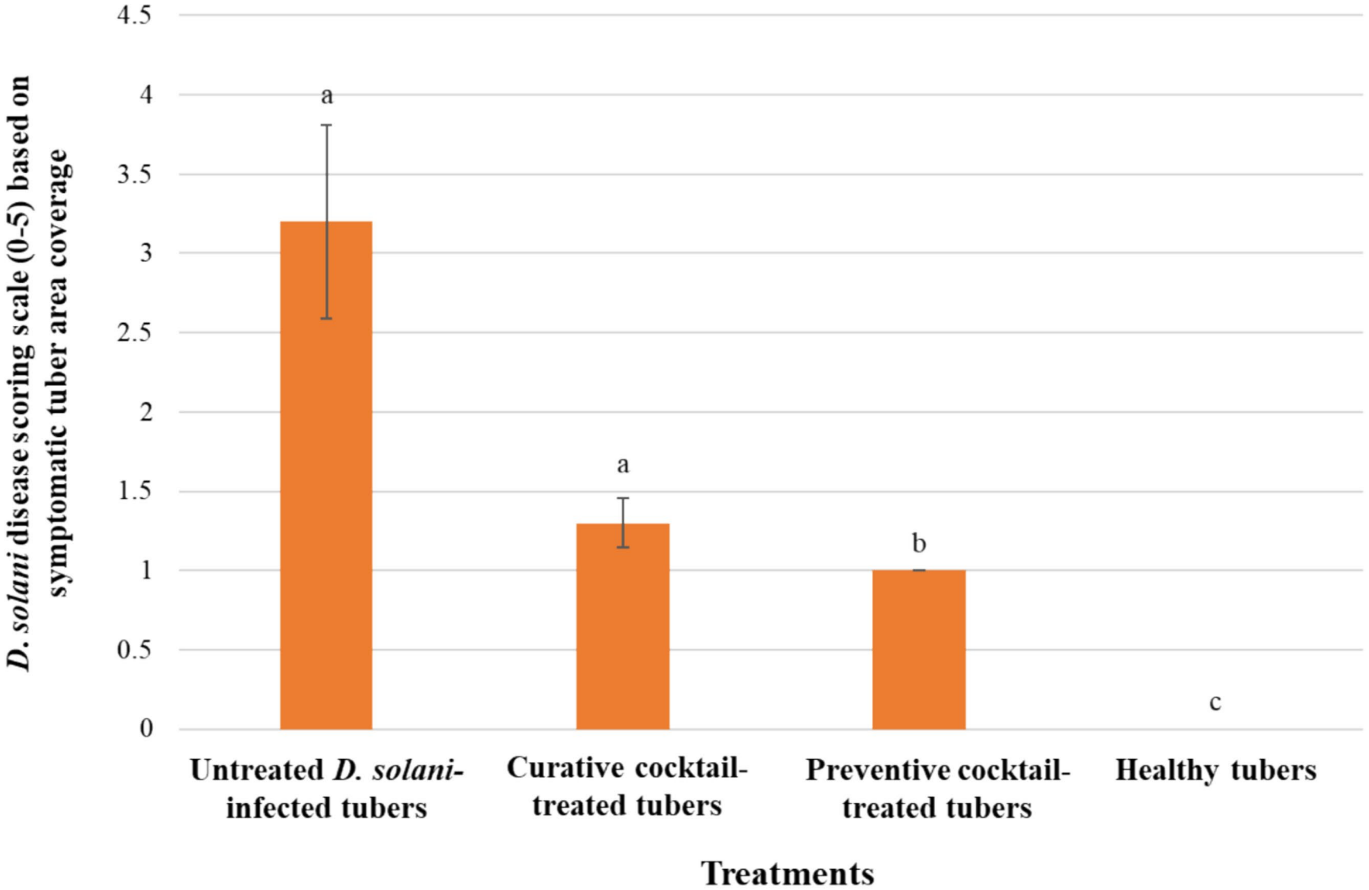
Histograms showing the severity of *Dickeya solani* symptoms on potato tubers treated preventively and curatively with the phage cocktail comprising LMST, PDS1, and PDS2. Error bars represent the standard error (SE) calculated from 10 biological replicates in a single experiment. A second independent experiment conducted under the same conditions produced similar trends (Supplementary Table 1), supporting the reproducibility of the results. Letters above the bars denote statistical groupings from a Dunn’s test (*p* < 0.05).

**Figure 9:**
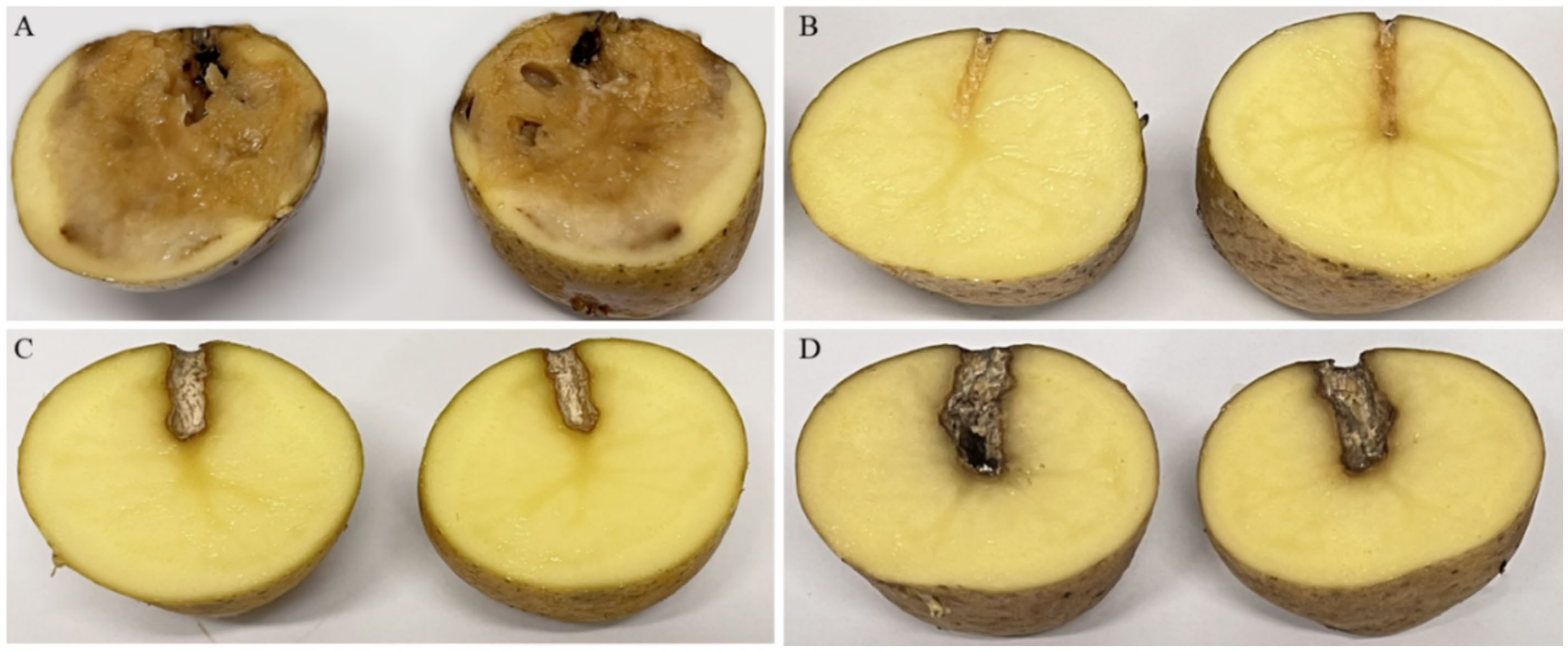
*Ex vivo* assays showing the biocontrol efficacy of the phage cocktail (LMST, PDS1, and PDS2) against *Dickeya solani* infections in potato tubers 7 dpi. **(A)** Untreated tubers inoculated with *D. solani*, exhibiting severe soft rot symptoms. **(B)** Healthy tubers serve as negative controls, showing no disease symptoms. **(C,D)** Tubers inoculated with *D. solani* and treated with the phage cocktail preventively **(C)** or curatively **(D)**, exhibiting mild disease symptoms.

**Figure 10:**
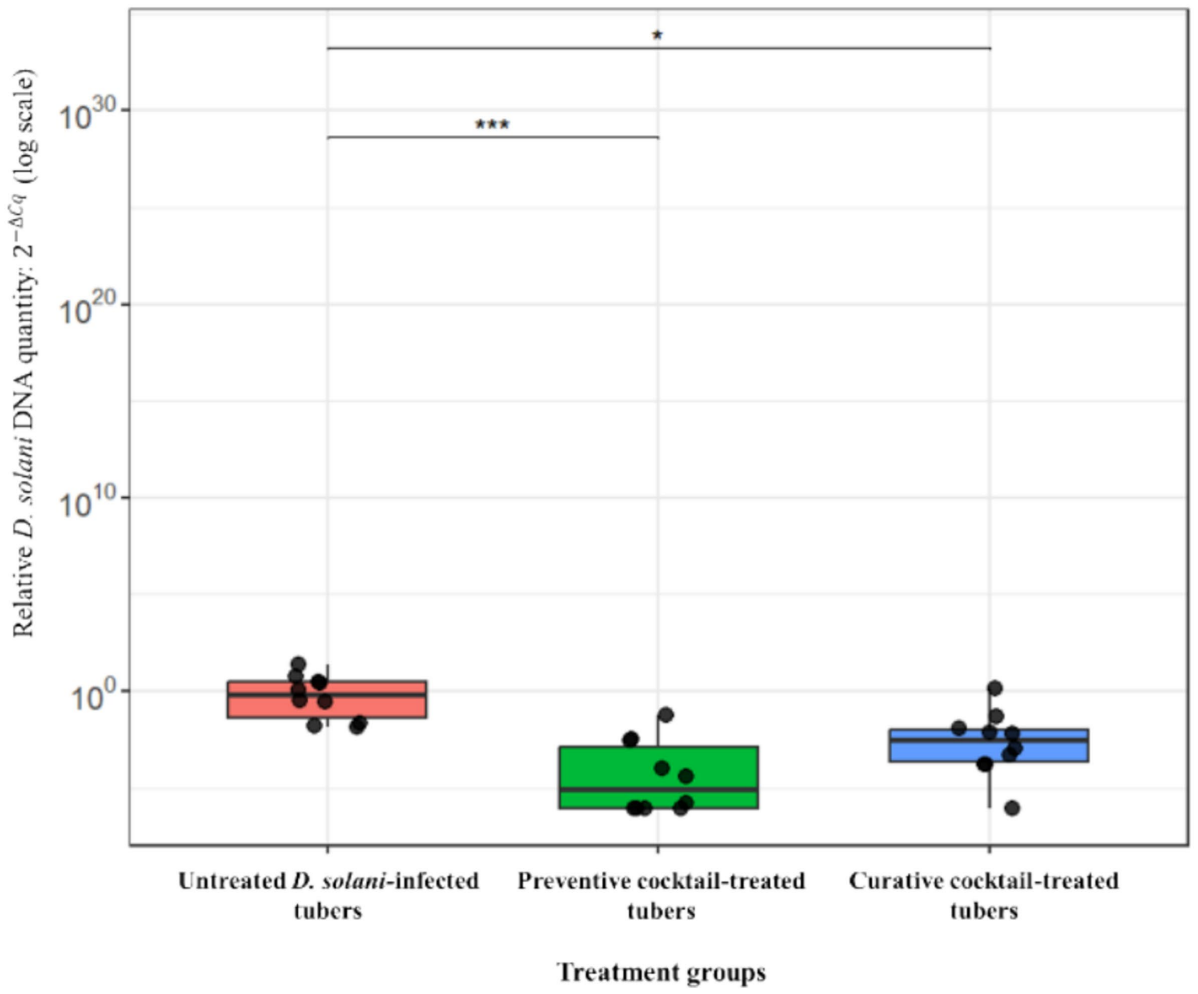
Box plot depicting the relative quantity of *Dickeya solani* DNA in potato tubers that were untreated or treated preventively and curatively with the phage cocktail. Asterisks denote statistically significant differences compared to the untreated control group (***p < 0.001, *p < 0.05).

The *ex vivo* application of the phage cocktail comprising LMST, PDS1, and PDS2 resulted in a significant reduction of soft rot symptoms caused by *D. solani* (*p* < 0.05) (Figure 8). In the absence of phage treatment, potato tubers inoculated with *D. solani* exhibited severe tissue maceration 7 days post-inoculation (dpi), with an average disease severity score of 3.2 (Figure 8). The infected tubers displayed extensive tissue maceration, discoloration, and water-soaked lesions that spread outward from the inoculation site (Figure 9). Conversely, tubers treated with the phage cocktail exhibited only slight tissue softening. In terms of efficacy, the preventive application of the phage cocktail resulted in a 68.75% reduction in disease severity, while the curative achieved yielded a 59% reduction in soft rot symptoms (Figure 8). These results were corroborated by TaqMan-based qPCR analysis, which demonstrated substantial differences in bacterial load between treated and untreated infected tubers (Supplementary Figure 2). The untreated control exhibited a mean quantification cycle (Cq) value of 20.92, closely resembling that of the *D. solani* positive control (10^8^ CFU/mL), which had a Cq of 19.98 (Supplementary Figure 2). In contrast, tubers treated with the phage cocktail displayed significantly delayed amplification, with mean Cq values of 34.75 and 29 for the preventive and curative treatments, respectively, indicating a substantial suppression of bacterial proliferation by the phage cocktail (Supplementary Figure 2). Furthermore, relative quantification of bacterial DNA demonstrated that the positive control group harbored significantly higher bacterial loads compared to both phage-treated groups (*p* < 0.001) (Figure 10). These molecular data corroborate the observed reductions in disease severity and provide compelling evidence that the phage cocktail exerts a potent biocontrol effect against *D. solani* in potato tubers.

## 4 Discussion

This study represents a significant contribution to the advancing field of bacteriophage-based biocontrol, providing insights into the rational design and development of phage strategies against phytopathogenic bacteria. Importantly, it addresses phage resistance, a fundamental constraint limiting both the efficacy and the broad-scale application of phages as sustainable biocontrol agents across diverse sectors. In this study, three bacteriophages (LMST, PDS1, and PDS2) were sequentially isolated to specifically target *D. solani* and its emergent phage-resistant strains. Rather than relying solely on taxonomic classification or genomic similarity of phages, the selection strategy focused on functional efficacy against resistant strains, enabling the construction of a phage cocktail with complementary host ranges. This rational design approach enhanced overall antibacterial activity and reduced the likelihood of resistance development, demonstrating a practical pipeline for the development of effective biocontrol agents. The three phages were isolated from wastewater, a proven reservoir for phage isolation owing to its rich bacterial density and diversity (Calero-Cáceres et al., 2019). Following a sequential isolation process, the obtained bacteriophages exhibited complementary infectivity against *D. solani* and its derived phage-resistant strains. Their formulation into a phage cocktail further restricted the adaptive potential of *D. solani* to evolve effective resistance, thereby reinforcing the robustness and long-term efficacy of the biocontrol strategy.

Spot assays showed that LMST-resistant strains of *D. solani* developed complete resistance to LMST, whereas PDS1- and PDS2-derived strains remained susceptible to their respective phages, indicating distinct resistance mechanisms. Remarkably, strains emerging from phage cocktail treatment retained susceptibility to all three phages, highlighting the efficacy of combinatorial phage pressure and suggesting a multiple-receptor hypothesis. Consistently, *in vitro* challenges of *D. solani* with LMST, PDS1, and PDS2, both individually and in combination under optimal conditions, demonstrated that the phage cocktail achieved superior suppression of bacterial growth compared to single-phage treatments and effectively mitigated the development of phage resistance. These results highlight the synergistic action of the three phages and indicate that simultaneous exposure imposes a combinatorial selective pressure that is difficult for *D. solani* to overcome. TEM analyses confirmed the lytic activity of LMST, PDS1, and PDS2, and notably revealed a resistance mechanism whereby *D. solani* produces OMVs that act as decoys to neutralize PDS2 phages. While OMV-mediated defense is well recognized in other bacterial systems (Egido et al., 2022), its demonstration in a phytopathogen like *D. solani* provides a novel insight into bacterium-phage interactions and significantly advances our understanding of phage resistance mechanisms in plant pathogenic bacteria. From a biocontrol perspective, these findings underscore the necessity of considering OMV-mediated defenses when designing phage cocktails, as phages with differing susceptibility to OMVs may be combined to maximize efficacy. This observation provides both mechanistic insight and practical guidance for the rational development of phage-based interventions against *D. solani*.

Phylogenetic analyses revealed that phages LMST and PDS1 cluster within the genera *Limestonevirus* and *Salmondvirus*, respectively, both of which include previously characterized phages infecting *D. solani*. This placement confirms their affiliation with established taxonomic groups and suggests that their biology and infection strategies may share commonalities with other members of these genera. In contrast, phage PDS2 displayed substantial divergence from known phage taxa, showing no significant sequence similarity to previously described viruses. This pronounced novelty indicates that PDS2 likely represents a new evolutionary lineage, thereby expanding both the genetic and functional diversity of phages available for targeting *D. solani*. The identification of such a divergent phage underscores the importance of exploring under-characterized phage populations to discover novel agents with potentially unique mechanisms of bacterial infection and lysis. Genomic analyses suggested their suitability as biocontrol agents, by confirming the absence of genes associated with lysogeny, virulence, or antibiotic resistance, which is an essential prerequisite for the safe applications of phages (Jones et al., 2012). Furthermore, host range analyses revealed that the phages exhibited activity primarily against *D. solani* strains, with no detectable effects on the tested beneficial bacteria. Notably, LMST was also able to infect *D. fangzhongdai*, which, to our knowledge, represents the first reported bacteriophage against this emerging species, recently identified in Italy (Sabri et al., 2025). This finding identifies LMST as a promising new candidate for the biocontrol of *D. fangzhongdai*. Additionally, thermal stability assays demonstrated that all three phages retained infectivity across a broad temperature range (−20 °C to 60 °C). Collectively, these findings underscore the suitability of the isolated phages as promising and safe biocontrol agents against *D. solani* infections. In this context, the outcomes of the *ex vivo* assays corroborated the *in vitro* findings, demonstrating a significant biocontrol efficacy of the phage cocktail against *D. solani* in potato tubers. Preventive and curative applications of the phage cocktail resulted in reductions of 68.75 and 59% in *D. solani*-induced symptom severity, respectively, and yielded a statistically significant decrease in bacterial load, as determined by TaqMan-based qPCR assays. These findings highlight the enhanced efficacy of prophylactic phage applications (Ahmadi et al., 2016; Skliros et al., 2023), suggesting that early intervention can more effectively suppress *D. solani* proliferation and disease progression. Our results are in agreement with previous studies on phage cocktails targeting soft rot pathogens; for example, Czajkowski et al. (2015) reported that a two-phage combination reduced *D. solani*-induced tuber maceration by over 80% on potato slices and exceeded 95% on whole tubers. Additionally, a multi-phage cocktail comprising ΦD1, ΦD2, ΦD3, ΦD4, ΦD5, ΦD7, ΦD9, ΦD10, and ΦD11 achieved a 30–70% reduction in soft rot incidence on potato slices (Czajkowski et al., 2014). Collectively, these data support the practical application of phage cocktails as effective strategies for controlling soft rot disease in potatoes.

This study reinforces the growing evidence that bacteriophage-based biocontrol offers an environmentally sustainable and effective strategy for managing *D. solani* infections, in line with the European Green Deal’s sustainability objectives. By rationally combining three distinct bacteriophages targeting *D. solani* and its resistant strains, we achieved enhanced antibacterial efficacy and minimized the development of phage resistance, highlighting the potential of this strategic cocktail design for sustainable disease control. This methodology provides a framework for future antibacterial strategies, highlighting the value of integrating resistance management into phage cocktail design to maximize efficacy and sustainability in plant disease control. Further investigations into *D. solani* phage receptors, resistance mechanisms, and associated fitness costs are warranted to optimize phage efficacy. Integrating phage therapy with complementary biocontrol agents such as bacteriocins or antagonistic bacteria could provide synergistic benefits. Additionally, the cloning and expression of lytic genes (e.g., endolysins and spanins) from LMST, PDS1, and PDS2 may enable the development of novel enzyme-based antibacterial formulations. Assessing cocktail stability under environmental stressors (pH, UV) and exploring pre-harvest applications (soil drenches, seed-tuber coatings) will be essential for ensuring robustness and broad applicability under agricultural conditions.

## Data Availability

The data presented in this study are publicly available. The data can be found at: https://www.ncbi.nlm.nih.gov, accession numbers PX290024, PX290025, and PX290026.
