# Supplementary material for: Tailored phage cocktail with resistance management for controlling Dickeya solani in potatoes
Source: Front Microbiol. 2026 Jan 27;17:1748314. doi: 10.3389/fmicb.2026.1748314 (PMC12886445; doi:10.3389/fmicb.2026.1748314)
Supplement: Supplementary file 1 [file Supplementary_file_1.docx]

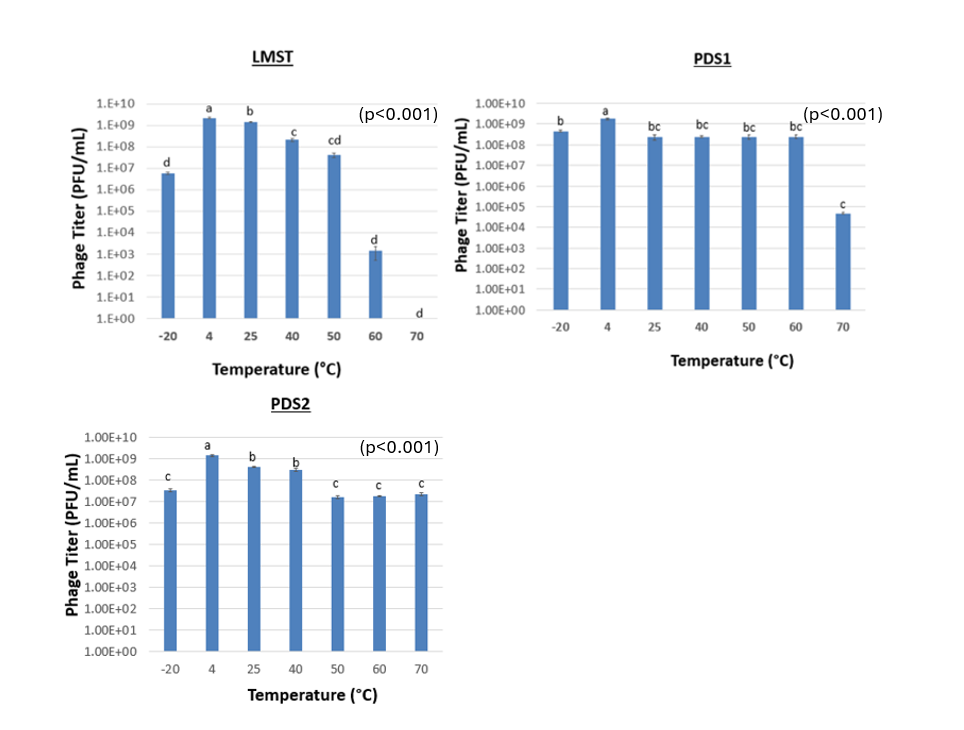


**Supplementary Fig. 1.** Histograms showing the thermal stability of *Dickeya* phages LMST, PDS1 and PDS2. Phage titers were determined using the double agar overlay method. Data represents the mean ± standard deviation of three biological replicates (*n* = 3). Different letters (a-d) above the bars indicate statistically significant differences (*p* < 0.001) between treatments.


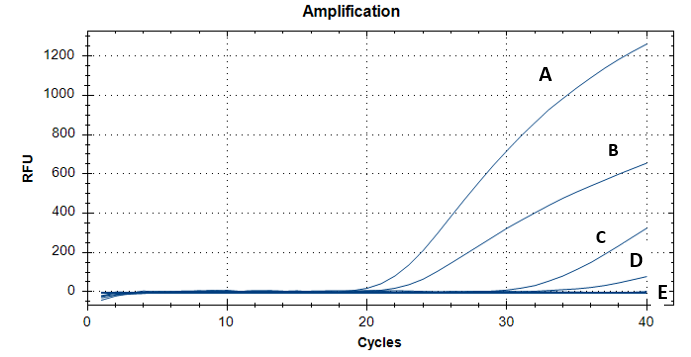


**Supplementary Fig. 2.** TaqMan-based qPCR assay showing representative DNA amplification curves obtained from: (A) Genomic DNA of *Dickeya solani* (10⁸ CFU/mL), used as a positive control reaction. (B) Genomic DNA extracted from untreated potato tubers infected with *D. solani*. (C, D) Genomic DNA extracted from potato tubers infected with *D. solani* and treated preventively (C) and curatively (D) with the phage cocktail. (E). Sterile water is used as a negative control.

**Supplementary Table 1**. Distribution of soft rot disease severity scores (scale 0–5) on potato tubers subjected to preventive and curative treatments with a phage cocktail consisting of LMST, PDS1, and PDS2, as assessed in the second experiment.

| Treatment-**Scale** | **0** | **1** | **2** | **3** | **4** | **5** |
| --- | --- | --- | --- | --- | --- | --- |
| Control (+) | 0 | 0 | 2 | 3 | 0 | 5 |
| Cocktail (preventive) | 0 | 0 | 10 | 0 | 0 | 0 |
| Cocktail (curative) | 0 | 0 | 6 | 3 | 1 | 0 |
